# Molecular Biomarkers and Influential Factors of Denitrification in a Full-Scale Biological Nitrogen Removal Plant

**DOI:** 10.3390/microorganisms8010011

**Published:** 2019-12-19

**Authors:** Hua Fang, Betty H Olson, Pitiporn Asvapathanagul, Tongzhou Wang, Raymond Tsai, Diego Rosso

**Affiliations:** 1 School of Environmental Science and Engineering, Nanjing University of Information Science & Technology, Nanjing 210044, China; fanghua@nuist.edu.cn; 2 Department of Civil and Environmental Engineering, University of California, Irvine, CA 92697, USA; bidui@uci.edu; 3 Water-Energy Nexus Center, University of California, Irvine, CA 92697, USA; 4 Department of Civil Engineering and Construction Engineering Management, California State University, Long Beach, CA 90840, USA; Pitiporn.Asvapathanagul@csulb.edu (P.A.);; 5 Los Angeles County Sanitation District, Carson, CA 90745, USA; astrellazero@gmail.com

**Keywords:** denitrification, nitrogen removal, full-scale activated sludge, functional genes, 16S rRNA gene

## Abstract

Three denitrifying bacteria, *Paracoccus* spp., *Thauera* spp., *Pseudomonas*-like spp., and two functional genes, nitrate reductase (*nar*G and *nap*A), were studied as potential biomarkers for total nitrogen removal. These bacterial genera and the functional genes showed significant negative correlations with total nitrogen in the effluent (TN_eff_). *Thauera* spp. had the highest correlation (*r* = −0.793, *p* < 0.001) with TN_eff_, and *nar*G-like and *nap*A genes also showed significant correlations (*r* = −0.663 and −0.643, respectively), suggesting functional genes have equal validity to 16S rRNA genes in monitoring denitrification performance. The most explanatory variables were a combination of constituents, with temperature emerging as the most important in Pearson’s correlation and redundancy analysis. *Thauera* spp. had the highest correlation with temperature (*r* = 0.739) followed closely by *Paracoccus* spp. (*r* = 0.705). Denitrification was also significantly affected by pH (*r* = 0.369), solids retention time (*r* = −0.377), total nitrogen_in_ (*r* = 0.635), and organic matter in the influent (biochemical oxygen demand and chemical oxygen demand; *r* = 0.320 and 0.522, respectively). Our data verified that major denitrifiers’ 16S rRNA genes and nitrate reductase genes were better biomarkers than the biomass concentration, and any of the biomarkers could track denitrification in real time.

## 1. Introduction

In biological nitrogen removal (BNR) processes, nitrogen conversion relies on biological metabolism, and a number of physicochemical parameters have been found to affect the performance of BNR systems. For example, dissolved oxygen (DO) in the anoxic zone, pH, mixed liquor temperature, mixed liquor suspended solids concentration (MLSS), availability of biodegradable carbon, carbon to nitrogen ratio (C:N ratio), and toxicity of the influent have an impact on nitrogen removal efficiency [1,2,3]. Amongst these operational conditions, MLSS have been used to estimate the amount of biomass and served as a surrogate to design process configurations and to predict process outcomes through activated sludge models. Nevertheless, MLSS are less indicative of active biomass in the activated sludge, especially in BNR processes [4].

With the advancement of molecular techniques, it is possible to derive the specific-microbial group abundance from the copies of 16S rRNA or functional genes, instead of MLSS concentrations. Many researchers have compared molecular findings of 16S rRNA or functional genes for ammonia oxidation and nitrite oxidation, hence transforming molecular data into a form used by the nitrification engineering profession [5,6]. In contrast with nitrification, molecular approaches to determine biomarkers in the denitrification process are scant in the activated sludge. This is probably because of the diverse phylogenic nature of denitrifying bacteria. Until now, nearly 130 bacterial species within more than 50 genera were identified as denitrifying bacteria [7]. Since engineering practices of wastewater treatment are normally based on overall process function, it may be better described by using predominant organisms responsible for denitrification as opposed to identifying all bacteria containing a suite of denitrification genes.

Several studies suggested that *Proteobacteria* was the dominant phylum within the activated sludge process and most likely responsible for denitrification (inter alia, [8]). The genera *Thauera* (*Betaproteobacteria*), *Paracoccus* (*Alphaproteobacteria*), and *Pseudomonas* (*Gammaproteobacteria*) were frequent denitrifying bacteria observed in the activated sludge [9,10,11]. Investigating these identified denitrifying genera is warranted in full-scale treatment plants. Nonetheless, 16S rRNA gene-based information may not accurately describe the bacterial biochemical level because denitrification is a generalized process, and genetic divergence within the functional genes that encode various steps is quite high [12]. Accordingly, this suggests using a functional gene, which corresponds with one step in the process, as an indicator of overall denitrification may be a more accurate way to assess total denitrifiers.

In denitrification, nitrate reductase is the first enzyme in the pathway. The membrane-bound nitrate reductase (*nar*G) and the periplasmic-bound nitrate reductase (*nap*A) are recognized to be widely dispersed through genera belonging to the Bacteria and Archaea kingdoms [13]. However, these functional gene biomarkers for nitrate reduction have not been studied in activated sludge samples due to difficulties in developing a universal assay target for the entire denitrifying group or functional genes responsible for encoding nitrate reduction [13].

The primary aims of this study were thus two-fold: (1) to investigate the major known denitrifying group and its relationship with the denitrification performance for identifying a valuable denitrification molecular biomarker; and (2) to understand the interaction between denitrifying bacterial dynamics and physicochemical parameters combined with operational factors for providing insight into how to improve denitrification performance. To this end, we quantified three well-studied denitrifying genera and two functional genes involved in the first step of the denitrification pathway via quantitative polymerase chain reaction (qPCR), and concurrently monitored operational or environmental variables over an 11-month period at a full-scale biological nitrogen removal plant (BNP) in Southern California. Multivariate statistical tools were applied to reveal relationships among nitrogen removal performance, denitrifiers, wastewater characteristics, and operational conditions.

## 2. Materials and Methods

### 2.1. Study Site and Wastewater Sample Collection

The wastewater samples were collected from the Michelson water reclamation plant (MWRP) having a capacity of 18 million gallons per day (MGD) or 68,137 cubic meters per day (m^3^/d), located in Irvine, California. Primary effluent underwent biological treatment for carbon and nitrogen compound removal in the activated sludge process. The plant consisted of both anoxic and aerobic zones designed for denitrification and nitrification, respectively. The plant was operated as a Modified Ludzack Ettinger (MLE) process with methanol addition for achieving nitrate removal.

Samples were taken from four different locations in the plant, including (1) primary effluent (i.e., secondary influent), (2) anoxic zone, (3) aeration tank, and (4) secondary effluent. Approximately 250 mL of samples were collected in sterile bottles on a weekly or biweekly basis from January through November. The samples were stored on ice and refrigerated at 4 °C until analyzed, within 24 h.

### 2.2. Bacterial Pure Cultures, Culture Media, and Growth Conditions

*Paracoccus denitrificans* (B-3785) and *Pseudomonas putida* (B-1023) pure cultures were kindly provided by the Agricultural Research Service, United States Department of Agriculture (USDA, Peoria, IL, USA). The cultures were grown in a medium recommended by the USDA. The *Escherichia coli* O157:H7 (ATCC 43895) culture was provided by the Food and Drug Administration: Pacific Regional Laboratory-Southwest (FDA, Irvine, CA, USA). *E. coli* growth condition was as described previously [14].

### 2.3. DNA Extraction

Total nucleic acids were extracted from three subsamples of each environmental grab sample using a modified bead beating protocol [14,15], which was fully described in Asvapathanagul et al. [16]. All DNA extracts were measured for concentration and purity (A260/A280) using a spectrophotometer DU^®^7400 spectrophotometry (Beckman, Orange, CA, USA). The pure cultures followed the same DNA extraction procedure as described for environmental samples. However, no triplicate analysis was performed, and cell lysis was conducted once.

### 2.4. PCR and qPCR Assays

All qPCR reactions, except total bacteria qPCR, were run using Eppendorf Realplex EP (Eppendorf, Hauppauge, New York, NY, USA). The total bacteria qPCR was described in previous work [17]. All primers and probes used in this study were listed in Table 1. The qPCR master mixture and qPCR condition for all real-time PCR assays were followed according to Tsai [18] and Wang [19]. The minimum detection limit of all three 16s rRNA qPCR assay was 5000 copies per reaction. For *Paracoccus* spp., *Pseudomonas* spp.-like, and *Thauera* spp. quantification, the average efficiency of qPCR runs was 0.99, 0.98, and 0.98, respectively, the mean coefficient of variation (R^2^) was 0.998, 0.994, and 0.991, respectively, and the average slope was −3.31, −3.37, and −3.47, respectively. For real time quantification of *nar*G-like and *nap*A genes using SYBR Green, the average efficiency was 0.99. The mean R^2^ was 0.998 and 0.996 with a slope of −3.34 and −3.40 for *nar*G-like and *nap*A, respectively. The minimum detection limit of the functional gene assays was 1000 copies per reaction. Melting curves were also conducted after the completion of the PCR cycle sequence. In all qPCR experiments, negative controls containing no template DNA were subjected to the same procedure. For PCR assays, the same temperature profile and master mixture without probe addition were used for each primer set.

### 2.5. Analysis of PCR Products, PCR Product Purification, and DNA Sequencing

Gel electrophoresis was conducted after PCR to confirm the amplicon size of PCR products. The sequencing was performed for both 3′–5′ and 5′–3′ strands, and the consensus sequences were included in the sequencing analysis using published software [23,24]. The protocols for gel electrophoresis, target bands purification, sequencing analysis, and fragment alignment were described previously [16].

### 2.6. Melt Curves

Melt Curve analysis was performed using Eppendorf RealPlex EP (Eppendorf, Hauppauge, New York, NY, USA) with temperature ramped from 55 to 99 °C in one-degree increments.

### 2.7. Standards for Real-Time PCR

The entire genomic DNA extract of *Paracoccus denitrificans* (B-3785), *Pseudomonas putida* (B-1023), and *E. coli* O157:H7 (ATCC 43895) were employed as standards for 16s rRNA gene quantification for *Paracoccus* spp., *Pseudomonas* spp.-like, and total bacteria, respectively. The qPCR standards for *Thauera* spp. and two functional genes were obtained from the amplified target amplicon of DNA extract from activated sludge using the primers indicated from Table 1.

### 2.8. Real-Time PCR Result Calculation

The conversion factors from 16S rRNA genes and functional genes to cells are listed in Table 2. The *Thauera* spp., *nar*G-like, and *nap*A gene abundance was quantified using each fragment as the standard. The qPCR results were then transformed as described in Asvapathanagul et al. [17] to represent quantification using the target fragments contained in a plasmid as the standard. The correlations of fragment and plasmid of *Thauera* spp., *nar*G-like, and *nap*A genes were *r* = 0.99.

### 2.9. Physical and Chemical Analysis

Temperature, dissolved oxygen concentration (DO), and pH were measured at the point of sample collection using DO and pH meters (HACH, Loveland, CO, USA). Other physicochemical and operational parameters, including biochemical oxygen demand (BOD), chemical oxygen demand (COD), ammonium ion (NH_4_^+^-N), total nitrogen (TN), methanol concentrations, mixed liquor suspended solids (MLSS), solids retention time (SRT), and hydraulic retention time (HRT), were obtained from laboratory personnel and plant operators in accordance with standard methods [27]. Food to biomass ratio (F/M), organic to nitrogen ratio (COD/TN), biochemical oxygen demand to chemical oxygen demand ratio (BOD/COD), and specific resource availability of nitrogen (Rs/X-N, Ginige et al. [28]), i.e., TN of secondary influent to MLSS ratio, were derived from measurable physical-chemical parameters, simultaneously.

### 2.10. Statistical Analysis

To test the correlation between two variables, Pearson’s correlations (*r*) were calculated using SPSS 20 (IBM, Somers, New York, NY, USA), and significance values (*p*) associated with correlation coefficients were calculated by the T-distribution function test (TDIST: two-tailed). To elucidate the interaction among denitrifying bacteria and nitrogen removal performance, as well as physicochemical and operational parameters, two multivariate statistical methods were performed. Principal component analysis (PCA) was applied to reveal any statistically significant relationship between denitrifier communities and nitrogen removal performance in an unconstrained ordination. Manual forward selection with the Monte Carlo permutation test was performed to determine the significance of the environmental variables with 499 permutations. PCA and redundancy analysis (RDA) were conducted by Canoco for windows version 4.53 (Plant Research international, Wageningen, Netherlands).

## 3. Results

### 3.1. Denitrifying Bacteria and Denitrifying Genes in this Study

**Figure 1:**
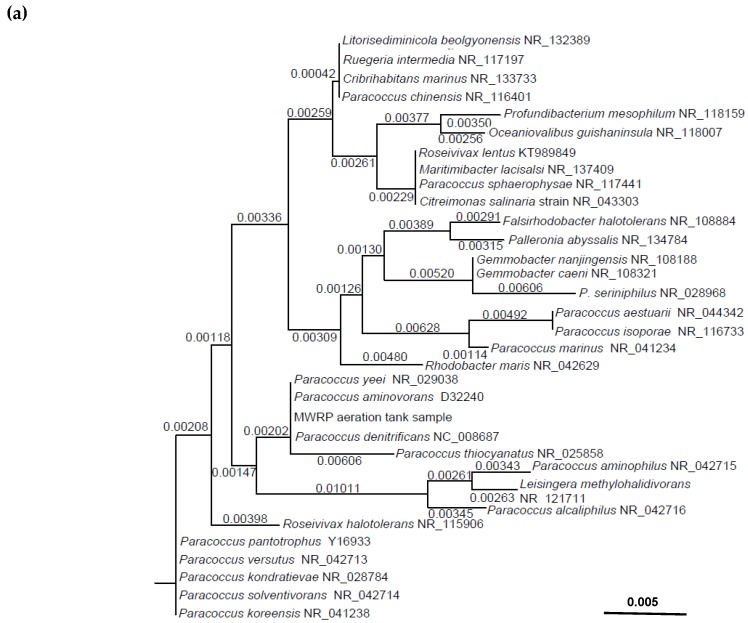

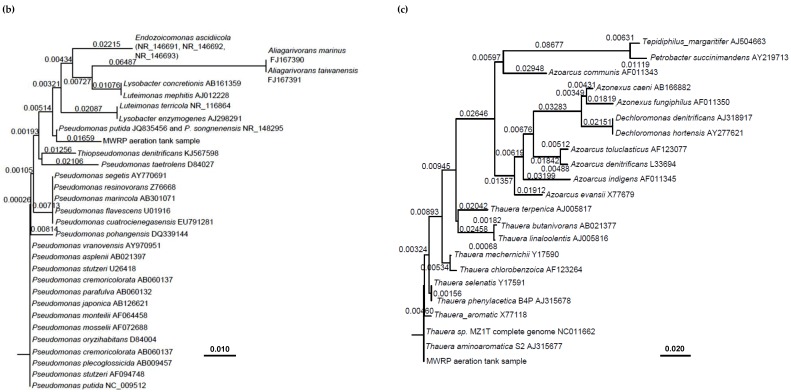

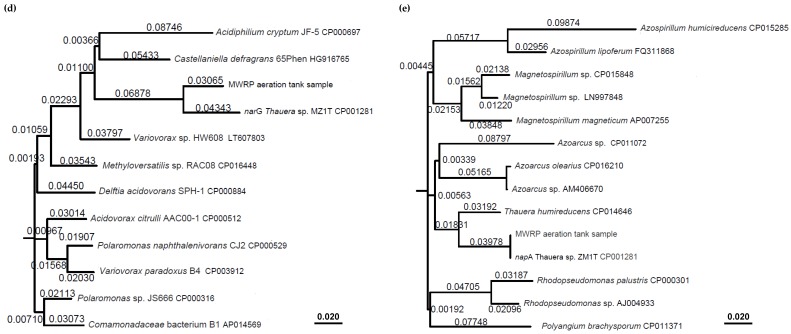
Phylogenetic analysis of the target sequences for 16S rRNA gene and functional genes (*nar*G and *nap*A) of the Michelson water reclamation plant (MWRP) aeration tank mixed liquor sequencing results compared to other target organisms and other bacteria. (**a**) *Paracoccus* spp., (**b**) *Pseudomonas* spp., (**c**) *Thauera* spp., (**d**) *nar*G and (**e**) *nap*A. The numbers above the branches indicate the dissimilarity among the sequences. GenBank accession numbers are displayed at the end. The scale bars are varied from Figure 1a–e. For Figure 1a, the scale bar indicates 0.005 inferred 5 nucleic acid base pair differences in 1000 nucleic acid base pairs. Remark: 7 *Paracoccus* species sharing 100% to the target sequence: *P. alcaliphilus* (KT261124), *P. aminovorans*, *P. denitrificans*, *P. huijuniae*, *P. siganidrum*, *P. thiocyanatus*, *P. yeei*.

The MWRP amplicons produced using the *Paracoccus*-like primers in this study displayed 100% similarity to the 165 basepair (bp) target sequence (*P. denitrificans*) (Figure 1a). This indicated the organisms amplified by this primer set were *Paracoccus* spp. Meanwhile, the 119 bp MWRP amplicons yielded by the *Pseudomonas* primer set, targeting organisms in genus *Pseudomonas*, showed highest (98.3%) similarity to *P. putida* (JQ835456) and *P. songnenensis* (NR_148295) (Figure 1b). Consequently, *Pseudomonas* spp.-like bacteria was denoted as the bacteria quantified using the *Pseudomonas* primer and probe set. Additionally, the 400 bp *Thauera* spp. target sequence obtained from *Thauera* sp. MZ1T shared 100% similarity to *T. aminoaromatica* (AJ315677) and the MWRP sequence found in our study (Figure 1c).

The 135 bp amplicons obtained from the *nar*G primer set amplification had 92.6% similarity to the *nar*G gene of *Thauera* sp. MZ1T. Hence, *nar*G-like genes were represented as cells quantified from the MWRP samples using the *nar*G primer set (Figure 1d). For the *nap*A gene amplification, the MWRP aeration tank sequence had 100% similarity to the 265 bp target for *nap*A gene of *T.* ZM1T (CP001281), the target bacteria (Figure 1e).

### 3.2. Operational Conditions, Wastewater Characteristics, and Bioreactor Performance

**Figure 2:**
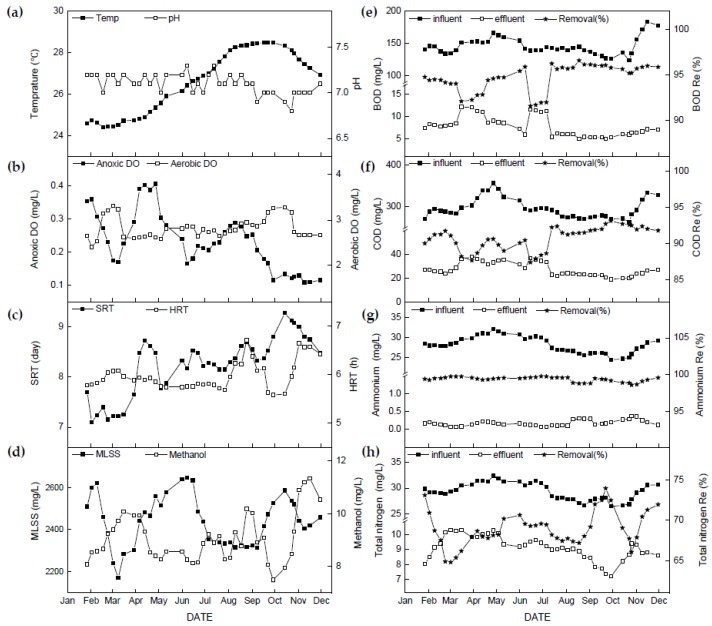
Variations in operational conditions and performance during the study period (**a**) temperature and pH; (**b**) anoxic and aeration tank dissolved oxygen concentration (DO); (**c**) solids retention time (SRT) and hydraulic retention time (HRT), (**d**) mixed liquor suspended solids (MLSS) and methanol dosing; (**e**) influent and effluent biochemical oxygen demand (BOD) and BOD removal; (**f**) influent and effluent chemical oxygen demand (COD) and COD removal; (**g**) influent and effluent ammonium and ammonium removal; (**h**) influent and effluent total nitrogen and total nitrogen removal. Remark: for (**e**) to (**h**) closed square indicating influent (primary effluent), open square indicating effluent (secondary effluent), and star indicating removal.

Operational and environmental parameters as well as bioreactor performance of MWRP over the study period are shown in Table 3 and Figure 2a–d. The mixed liquor temperature varied from a winter minimum of 24.4 °C to a summer maximum of 28.5 °C. The effluent pH remained in a narrow range with an average of 7.1 ± 0.11. The mean of dissolved oxygen in the anoxic zone (DO_anoxic_) was 0.23 mg/L, but showed substantial oscillations (0.12–0.41 mg/L) from January to September, after which it remained less than 0.2 mg/L. By contrast, DO in the aerobic zone (DO_aerobic_) was maintained at 2.82 ± 0.23 mg/L for the entire study period. Solids retention time (SRT) showed a gradually increasing trend and fluctuated over a wide range (7.1–9.3 days). Hydraulic retention time (HRT) was stable from January to July but varied during the later period of the study. Mixed liquor suspended solids concentrations (MLSS) with an average of 2437 ± 119 mg/L and methanol dosing with average of 9.06 ± 0.92 mg/L also showed oscillatory fluctuations over one year.

The performance of the full-scale plant was stable throughout the study (Figure 2e–h and Table 3). The effluent concentrations and the removal rates with averages of 8 mg/L and 94.7% for biochemical oxygen demand (BOD), and 27 mg/L and 90.8% for chemical oxygen demand (COD), respectively, followed similar trends and maintained high carbon removal performance. Influent ammonium ion (NH_4_^+^-N) level (NH_4_^+^-N_in_, 28.17 ± 2.08 mg/L) and influent total nitrogen (TN) level (TN_in_, 29.36 ± 1.68 mg/L) were very close, with approximately 95% of TN composed of NH_4_^+^-N within the secondary influent. Effluent level and removal rate of NH_4_^+^-N with an average of 0.18 mg/L and 99.4% were nearly constant, which showed nitrification performance at this BNR plant was considerably stable across the study campaign. Effluent and removal rates of TN, with an average of 9.12 mg/L and 68.9%, fluctuated over a relatively wide range, indicating the unsteadiness of the denitrification performance of the BNR system.

The Pearson’s correlation coefficients (*r*) between operational or environmental variables, which were assumed to be significant drivers of TN removal, and concentration of effluent TN (TN_eff_) are also listed in Table 3. Temperature (*r* = −0.676, *p* < 0.01), NH_4_^+^-N_in_ (*r* = 0.700, *p* < 0.01) and TN_in_ (*r* = 0.635, *p* < 0.01) showed a strong significant correlation with the level of TN_eff_. pH (*r* = 0.369, *p* < 0.05), DO_anoxic_ (*r* = 0.361, *p* < 0.05), SRT (*r* = −0.377, *p* < 0.05), and BOD_in_ and COD_in_ (*r* = 0.320, *p* < 0.05 and *r* = 0.522, *p* < 0.01, respectively) also showed significant correlations with TN_eff_. Contrary to expectation, MLSS and methanol dosing did not correlate to TN_eff_ (*p* > 0.05).

### 3.3. Temporal Dynamics of Denitrifying Bacteria and Functional Genes

**Figure 3:**
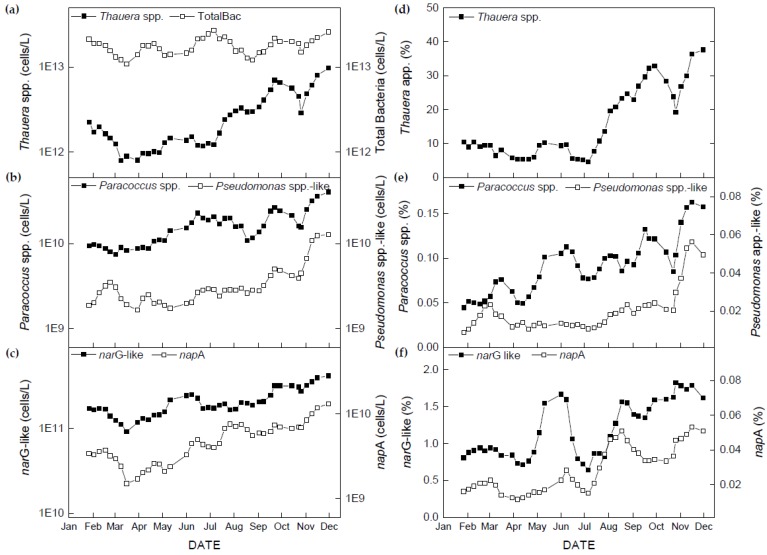
Changes in quantities of bacteria and functional genes measured in this study (**a**) total bacteria and *Thauera* spp.; (**b**) *Paracoccus* spp. and *Pseudomonas* spp.-like; (**c**) *nar*G-like and *nap*A genes; and changes in relative proportions to total bacterial abundance of (**d**) *Thauera* spp.; (**e**) *Paracoccus* spp. and *Pseudomonas* spp.-like; (**f**) *nar*G-like and *nap*A genes in the full-scale plant during the study period.

For forty activated sludge samples collected from MWRP, 16S rRNA genes for total bacteria and three denitrifying genera (*Thauera*, *Paracoccus* and *Pseudomonas*-like), and two nitrate reduction functional genes (*nar*G-like and *nap*A) were quantified via qPCR assays. Temporal dynamics for total bacteria, three denitrifying genera and two functional genes, as well as their relative abundance (%) to total bacteria are illustrated in Figure 3. Average quantities of each bacterial and functional gene groups are also shown in Table 4.

The total bacterial population with the average of 1.80 × 10^13^ cells/L fluctuated in a narrow range during the entire study period. The amount of *Thauera* spp. (average of 2.86 × 10^12^ cells/L) decreased from January (2.23 × 10^12^ cells/L) to March (9.57 × 10^11^ cells/L), and then was stable until June, after which it increased to 9.72 × 10^12^ cells/L until the end of the study with the exception of October (Figure 3a). The relative abundance of *Thauera* spp. remained constant (5.34%−10.45%) in earlier stages of study and rose quickly (up to 37.60%) in the later period, except for October (19.27%) (Figure 3d). The amount of *Paracoccus* spp. and *Pseudomonas-*like bacteria were two and three orders of magnitude less than the amount of *Thauera* spp., and behaved by gradually increasing during the study period, representing an average of 0.09% and 0.02% of total bacteria, respectively (Figure 3b,e). Among the three denitrifiers, *Thauera* spp. was the major denitrifying lineage, but not more than 50% of total bacteria (Figure 3d). The changes in pattern in the abundance of bacteria containing the two functional genes, *nar*G-like and *nap*A, were similar to that of denitrifiers, with averages of 2.10 × 10^11^ and 5.14 × 10^9^ cells/L, respectively. The *nar*G-like gene abundance was approximately 2 orders of magnitude higher than that of *nap*A gene.

The correlation between denitrifying bacteria and TN_eff_ are listed in Table 4. The population of *Thauera* spp. cell abundance showed the strongest negative correlation (*r* = −0.793, *p* < 0.001) with TN_eff_, and the other two denitrifiers and two functional genes also showed strong significant inverse correlation to TN_eff_. Only total bacteria had a weak significant relationship with TN_eff_. Table 5 displays high correlation among all denitrifying bacterial population and functional genes in this study.

**Figure 4:**
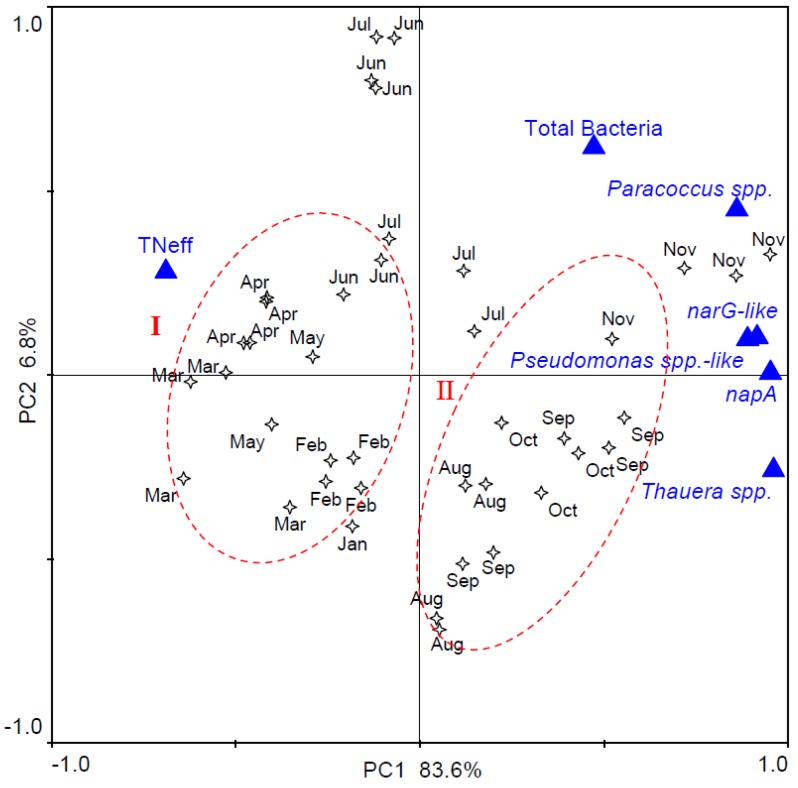
Principal component analysis (PCA) biplot of denitrifiers and nitrogen removal performance. Asterisks represent sampling date, and triangles indicate bacterial or gene abundance.

To further explore relationships between denitrifying biomarkers and denitrification performance, the quantities of three denitrifying bacteria, total bacteria, two nitrate reduction functional genes, and TN_eff_ were assessed using PCA analysis, and the biplot is shown in Figure 4. The PC1 and PC2 accounted for 83.6% and 6.8% of total variance, respectively. All denitrifying bacteria and functional genes distributed along the positive PC1, whereas TN_eff_ was located at the negative of PC1 (Figure 4). This indicated that the amount of denitrifying bacteria and functional genes resulted in lower values of TN_eff_, especially *Thauera* spp. that displayed an opposite direction of TN_eff_. The total bacteria abundance was close to PC2, which implied it did not significantly associate with nitrogen removal. Although the samples, indicated by stars, spread over the various phases of the biplot, the majority of the samples could be categorized into two groups (I and II) according to the sampling date. Group I and II represented winter & spring (January to June) and summer & fall (from July to November), respectively. Samples collected at similar time periods were located closely in the biplot, which inferred that the abundance of denitrifying bacteria and denitrification performance of the full-scale BNR plant were affected by seasonal factors, notably temperature.

### 3.4. Correlation between Denitrifier Abundance, Wastewater Characteristics, and Operational Conditions

In order to discern the possible correlation between denitrifying population, wastewater characteristics, and operational parameters, two parallel but complementary statistical approaches were employed in this study. Links between the abundance of denitrifying bacteria combined with functional genes and wastewater characteristics as well as operational conditions were initially explored via calculation of Pearson’s correlation coefficients (Table 6). All of three denitrifiers were significantly positively correlated to temperature, SRT, and BOD/COD, and significantly negatively correlated to pH and DO_anoxic_ simultaneously. MLSS had no relationship with any denitrifiers. Only *Thauera* spp. bacterial abundance showed strong inverse correlations to NH_4_^+^-N_in_, TN_in_, Rs/X-N and F/M. Methanol dosing and carbon to nitrogen ratio (C/N; COD/TN) were just associated with *Pseudomonas-*like bacterial cell abundance. Unexpectedly, total bacteria did not correlate to any of operational parameters. Two functional genes, *nar*G-like and *nap*A gene cell abundances, showed a close relationship with the majority of operational factors, as well as followed similar trends to *Thauera* spp. cell concentrations, implying similar co-variance.

**Figure 5:**
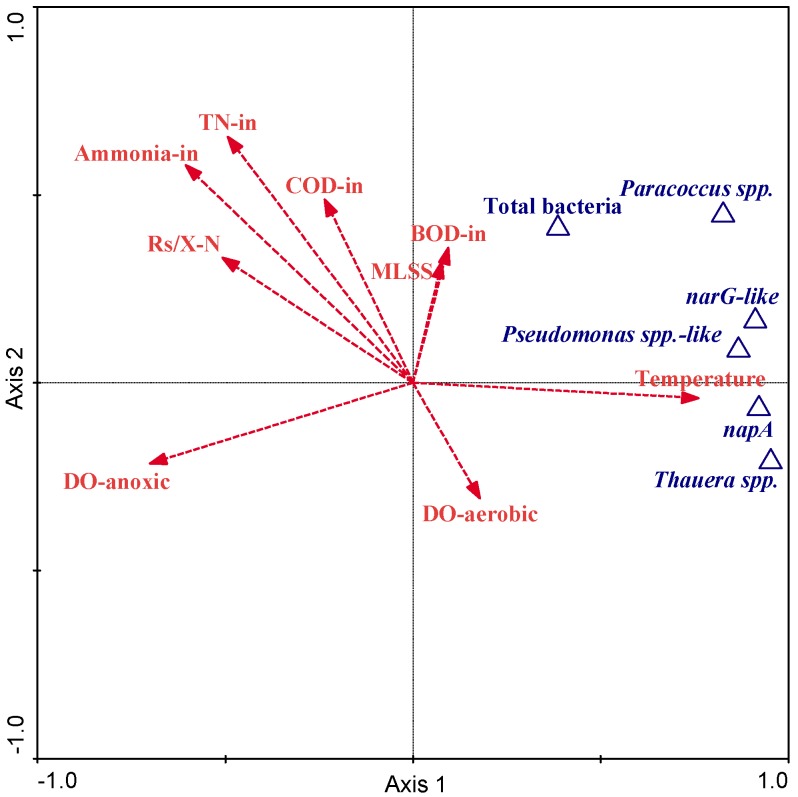
Redundancy analysis between denitrifier abundance and operational conditions. Triangles indicate bacterial and functional gene abundance. Arrows represent wastewater characteristics, operational parameters and environmental factors.

Correlations among the abundance of denitrifying bacteria and operational conditions were further evaluated via a direct gradient ordination method, redundancy analysis (RDA). In RDA ordination, the patterns of denitrifying bacteria were constrained by input environmental variables. The ordination explained most of the variance (92.5%) of genus-environment relationships and the majority of variance (85.8%) in genus. Both axes displayed high species-environment correlations (axis1: 0.98; axis 2: 0.91), indicating strong correlations between denitrifying bacterial abundance and operational conditions. Amongst 16 input environmental variables, nine were identified as significantly (*p* < 0.05) correlating to denitrifying bacterial abundance by RDA forward selection (Table 6). These nine variables were indicated in the ordination biplot (Figure 5) by arrows. Temperature showed the strongest correlation to the abundance of denitrifying bacteria, in agreement with the previously mentioned Pearson’s correlation coefficients. DO_anoxic_, NH_4_^+^-N_in_, and TN_in_ were identified as strong negatively correlated variables to denitrifier dynamics also in line with the previous result. Nevertheless, some operational factors, such as pH and SRT, which had strong significant relationships with denitrifier dynamics via Pearson’s correlation coefficients, were not involved in the final RDA ordination. This indicated variations of these factors were likely attributed to other more important operational factors during the study periods, and multivariate statistical methods facilitated identifying the real influential factors of bacteria dynamics in a full-scale Water Reclamation Plant (WRP).

## 4. Discussion

Denitrifying community structures varied significantly from one WRP to another due to treatment processes and operational conditions, but bacterial genera isolated from denitrifying bioreactors were closely related to species in *Proteobacteria* [8,29,30], including *Thauera* spp., *Paracoccus* spp., and *Pseudomonas* spp., for example. Long-term studies of *Thauera* spp. population in activated sludge are still limited, while many publications found *Thauera* spp. as a major denitrifier in activated sludge [31,32]. The amplicon sequence results and melt curve analysis from qPCR using *Thauera* spp. primer set, *Paracoccus*-like primer set, and *nap*A gene primer set confirmed the organisms quantified were *Thauera* spp., *Paracoccus* spp., and napA genes of *Thauera* sp. MZ1T in this study site (Figure 1). On the other hand, the amplicons produced from *Pseudomonas* spp. primer set and *nar*G primer set did not show 100% similarity to their target sequence (*Pseudomonas* spp. and *nar*G gene of *Thauera* sp. MZ1T, respectively). The high specificity of available *nar*G and *nap*A primers exhibited limited detection of other *nar*G and *nap*A groups [33,34]. Accordingly, there are several *nar*G and *nap*A primer sets published, and each was developed for specific groups of *nar*G and *nap*A genes in selected environments [35,36,37]. Our *nar*G amplicon sequencing result indicated the distingue *nar*G sequence in the activated sludge samples.

Michelson water reclamation plant (MWRP) is a typical domestic WRP. The process performance of the plant was generally efficient and reliable due to favorable environmental and operational conditions (Figure 2). In comparison to NH_4_^+^-N removal performance (NH_4_^+^-N_eff_: 0.18 ± 0.08 mg/L, NH_4_^+^-N Re%: 99.3% ± 0.3%), the performance of denitrification, i.e., TN_eff_ (9.12 ± 0.81 mg/L) and TN removal ratio (68.9% ± 2.17%), was more unstable (Figure 2). Nitrification is regarded as the rate-limiting step of BNR systems and is susceptible to environmental conditions in some previous research [38,39]. However, our findings indicate the performance of denitrification is likely more susceptible to environmental conditions than that of nitrification in this full-scale bioreactor and identifying those factors in the denitrification step can contribute to stability of nitrogen removal performance in BNR systems.

The denitrification performance of MWRP was affected by several conventional environmental and operational factors (Table 3). Whereas MLSS, which have been widely used for interpretation of nutrient removal processes for modeling work and process design [40], were not linked to the denitrification performance in our study. The parameter MLSS actually represented a lump-sum of the microbial community in activated sludge, as well as nonviable organic carbon and inert solids. Rittmann et al. [4] regarded MLSS as a poor input for purpose to predict the specific nutrient removal output of bioreactors. Our findings agreed with this point and suggested some novel parameters which were more suitable to monitor the nitrogen removal performance and should be developed as biomarkers in full-scale BNR systems.

The average of the total bacterial population in this study, quantified via qPCR assays, was 1.80 × 10^13^ cells/L, which remained relatively stable. The quantities were congruent with others research results from activated sludge samples [41,42]. Among the three targeted denitrifying bacteria, *Thauera* spp., which represents the *Betaproteobacteria*, accounted for approximately 16% of total bacteria on average and were much greater than *Paracoccus* spp. (*Alphaproteobacteria*, 0.09% on average) and *Pseudomonas*-like bacteria (*Gammaproteobacteria*, 0.02% on average) during the study period (Figure 3 and Table 4). Yet, these minor bacteria were always present over the study. Juretschko et al. [43] reported the abundance of the genus *Thauera* represented about 16% of the total bacteria using quantitative fluorescent in situ hybridization (FISH) in a nitrifying-denitrifying plant based upon a single sample, which highly agreed with our findings. Furthermore, Thomsen et al. [9] also found *Thauera* spp. quantified using FISH were the second or third richest group of denitrifying bacteria found from 16 municipal BNR plants with and without phosphorus removal in Denmark. Besides, several studies reported *Thauera* spp. was the dominant group in the microbial community of a full-scale WRP with nutrient removal [31,32]. Our findings were highly congruent with this research. Therefore, it was reasonable to conclude that the genus *Thauera* was a major denitrifying bacterium of MWRP.

*nar*G and *nap*A genes encode for nitrate reductase. *nar*G-like gene cell abundance was approximate 20 to 90 times more prevalent than *nap*A gene concentration depending on the sample date (Figure 3). In a related study, Bru et al. [35] found the predominant nitrate reductase in the soil and the freshwater environment was *nar*G. Our study agrees with their findings and extends this finding to the activated sludge in BNR plants. However, our quantities of *nar*G-like and *nap*A gene abundance were one order of magnitude higher than in the other environments tested by Bru et al. [35]. This may be due to our samples being from activated sludge operated for enhancing denitrification. Additionally, both nitrate reductase functional gene concentrations were highly correlated to the concentrations of 16S rRNA genes for *Thauera* spp., which may be attributable to the primer design because both functional genes of *Thauera* spp. were served as targets for the primer sets [18]. This indicated that nitrate reductase functional genes in this study mainly originated from *Betaproteobacteria*, although they were only a portion of total bacteria in activated sludge.

The abundance of three denitrifying bacteria and the two functional genes showed significant negative correlations with TN_eff_, while total bacteria had a weak link (Table 4). It turned out that these specific denitrifying microorganisms performed nitrate removal roles and could represent performance of nitrogen removal rather than total microorganisms in activated sludge. Within all denitrifying bacteria and functional genes tested in the study, the *Thauera* genus was found to have the highest correlation (*r* = −0.793, *p* < 1 × 10^−8^) with TN_eff_. This indicated the 16S rRNA gene abundance of *Thauera* spp., the major denitrifier in the MWRP, was the best indicator to track denitrification performance. Two nitrate reductase functional genes, *nar*G-like and *nap*A population in this study, also have been strong correlation (−0.663 and −0.643, respectively) with TN_eff_, which implied the functional genes biomarkers may have the equal validity to the 16S rRNA genes biomarkers in monitoring denitrification performance in this full-scale BNR plant.

Lu et al. [5] used two functional genes as biomarkers to determine the denitrification kinetics in the sequencing batch reactor. Their results suggested that using functional genes as biomarkers to trace the denitrification kinetics were possible as long as the predominant group that utilized the substrate was discovered. Our study was also based on the same concept. The finding of our study verified both the 16S rRNA genes of major denitrifiers and that major nitrate reductase functional genes were better biomarkers than MLSS to monitor the denitrification performance in this plant. The data can also be used in conjunction with traditional activated sludge models to improve the accuracy and stability of simulation results [1].

Denitrifying microbial community structure and function were controlled by a combination of factors. Within the operational and environmental variables determined in this study, temperature emerged in both Pearson’s correlation (*r*) and redundancy analysis (RDA) as the most important variable affecting the temporal dynamics of denitrifying bacteria. Denitrifying bacteria were mostly mesophilic [44], and the optimal temperature for wastewater denitrification is reported between 20 to 30 °C [45]. The MWRP activated sludge temperature was within this range. Consequently, the populations of denitrifying bacteria had a strong positive correlation with temperature in our study (Table 6).

Apart from temperature, dissolved oxygen in the anoxic zone (DO_anoxic_) was another important variable that affected growth of denitrifiers and performance of denitrification (Figure 5 and Table 6). In practice, DO_anoxic_ was generally supposed to be kept below 0.2 mg/L because oxygen can have an immediate inhibitory effect on denitrification [46,47]. Specifically, Hernandez and Rowe [48] indicated that expression and activity of nearly all nitrogen oxide reductases were suppressed in the presence of oxygen. All three denitrifying bacteria and two nitrate reductase functional genes were significantly associated with DO_anoxic_ in our study (adversely), which agreed with these findings. Pearson’s correlation analysis showed no correlation between denitrifying bacteria or functional genes and dissolved oxygen in the aerobic zone (DO_aerobic_), but DO_aerobic_ appeared to be the weakest significant factor using RDA (Table 6). However, the RDA analysis biplot demonstrated no influence of this parameter to the abundance of denitrifying bacteria and functional genes (Figure 5). Our findings suggested that the quantities and performance of denitrifying bacteria will noticeably benefit from keeping a stringent anoxic environment in denitrification process.

Many studies reported operating parameters, such as SRT and pH, not only result in long-term succession of community structure and dynamics, but also affect overall nitrogen removal [45,49]. The optimal pH for wastewater denitrification was from seven to nine [45]. The average of SRT and pH were 8.24 days and 7.10, respectively, during the study period, which were within the favorable range for denitrifiers growth. Unexpectedly, the two parameters showed no statistically significant relationship in RDA ordination (Table 6), although the three denitrifiers and two functional genes measured in this study correlated significantly via Pearson’s correlation. This was likely because RDA included all three denitrifiers, two functional genes, and total bacterial populations. The total bacteria variation caused no relationships to both pH or SRT via Pearson’s correlation. This might lower the degree of significance for pH and SRT variables in RDA analysis (Figure 5 and Table 6). Additionally, both SRT and pH correlated significantly to temperature via Pearson’s correlation coefficients (*r* = 0.72, *p* < 1 × 10^−7^; *r* = −0.453, *p* < 0.001) simultaneously. Accordingly, temperature, the most important influencing factor in the denitrification process, probably masked the small selection pressures imposed by SRT and pH on denitrifiers in multivariate statistical analysis.

Although methanol has been the most widely used as an extra carbon source for enhancing denitrification performance, only *Pseudomonas*-like bacteria were significantly correlated with methanol dosage in MWRP (Table 6). *Pseudomonas*, which was reported as a methanol utilizer in previous research [50], a minor denitrifying genus in this BNR system, only accounted for about 0.02% of total bacteria. This indicated adding methanol could not increase the quantities and capacity of denitrifiers in this plant. It was likely because the influent of MWRP belonged to domestic wastewater and contained enough carbon sources for the denitrification process. This finding, meanwhile, inferred that cutting down the dosage of methanol might be a feasible path to reduce operational costs at MWRP.

There was a substantial divergence for the relationship with nitrogen concentrations in the influent within the three denitrifiers. The concentration of influent ammonium ions (NH_4_^+^-N_in_) and total nitrogen (TN_in_) as well as specific resource availability of nitrogen (Rs/X-N) were significantly negatively correlated with *Thauera* spp. cell abundance but had zero or weak correlations with *Paracoccus* spp. and *Pseudomonas*-like bacteria (Table 6). This infers the *Thauera* genus tends to prevail in low nitrogen loading of influent. Ginige et al. [28] discovered that imposing a certain Rs/X ratio can influence the dominance of specific denitrifiers in activated sludge. When Rs/X ratio was low, i.e., the supply of nitrogen was restricted, *K* strategist of the studied denitrifiers dominated over others in the bioreactor because of their higher affinities towards resources. According to this finding, it is reasonable to assume that *Thauera* spp. can be classified as a *K* strategist, while *Paracoccus* spp. and *Pseudomonas-*like bacteria probably belong to *r* strategists. In comparison to industrial wastewater [38], the influent of the targeted WRP in this study is domestic wastewater, in which the concentration of nutrients is low and belongs to a resource restricted condition for microorganisms. This may be the reason that *Thauera* spp. overcomes others denitrifiers in the BNR system. In addition, denitrifiers with the same function and different eco-physiological characterization coexist in a bioreactor leading to functional redundancy of the denitrification system. System stability is better correlated with functional redundancy [51], which may be an important reason that MWRP behaved with stable nitrogen removal performance during the study period.

## 5. Conclusions

In conclusion, based on eleven-month monitoring in a full-scale BNR system, *Thauera* spp., as well as bacteria having functional genes *nar*G-like and *nap*A, showed strong significant correlations with the nitrogen removal performance. These molecular biomarkers were better indicators of the denitrification process than conventional parameters, such as MLSS. The activated sludge temperature and DO in the anoxic tank were the two most important operational conditions affecting the community dynamics of denitrifiers. Additionally, the specific resource availability of nitrogen in the influent could manipulate the population of denitrifiers with distinct survival strategies, which resulted in functional redundancy and stable denitrification performance in the full-scale BNR system.

## Figures and Tables

**Table 1:** Primers and probes used in this study.

Target Organisms/Genes	Primers and Probes	Sequence (5′–3′)	Annealing Temperature (°C)	Reference
***Paracoccus*-like spp.**	16S Para F	GGGCAGCATGCTGTTCGGTG	60.0	[18]
	16S Para R	GCGATGTCAAGGGTTGGTAA		
	16S Para-Taq	ACACCTAACGGATTAAGCATTCCGCCTGGG		
***Pseudomonas* spp.**	16S Pse F	GGATTGGTGCCTTCGGGAAC	60.1	[18]
	16S Pse R	ACGTGCTGGTAACTAAGGA		
	16S Pse-Taq	CACAGGTGCTGCATGGCTGTCGTCAGCTCG		
***Thauera* spp.**	16S Thau F	CGTGGTCTCTAACATAGGCC	66.9	[19]
	16S Thau R	CGGAGCAGCAATGCACTGAG		
	16S Thau-Taq	TACCGGACTAAGAAGCACCGGCTAACTACGT		
***nar*G**	NarG F	CAGGCGGCCGCGGATCATCGGG	66	[18]
	NarG R	CAGCAGACCGACTACCCGCGC		
***nap*A**	NapA F	CAGCCCATCGGCTCGTC	66	[18]
	NapA R	AGAACGGCGAGTTCACG		
**Total bacteria**	1055f	ATGGCTGTCGTCAGCT	50	[20]
	1392r	ACGGGCGGTGTGTAC		[21]
	16STaq1115	CAACGAGCGCAACCC		[22]

**Table 2:** Mean conversion factors for copies of 16S rRNA genes and functional genes to cells.

Groups	Copies/Cells	References
Total bacteria	3.6	[22]
*Paracoccus* spp.	3	[25]
*Thauera* spp.	4	[26]
*Pseudomonas* spp.-like	4.6	[25]
*nar*G-like gene	1	[26]
*nap*A gene	1	[26]

**Table 3:** Average operational parameters and bioreactor performance and Pearson’s correlation coefficient (*r*) with total nitrogen concentration in secondary effluent.

Parameters	Average	Standard Deviation	Pearson’s Correlation Coefficients (*r*) with TN_eff_
Temperature (°C)	26.6	1.51	−0.676 **
pH	7.1	0.11	0.369 *
DO_anoxic_ (mg/L)	0.23	0.09	0.361 *
DO_aerobic_ (mg/L)	2.82	0.23	−0.230
SRT (day)	8.24	0.59	−0.377 *
HRT (h)	5.98	0.29	−0.020
MLSS (mg/L)	2437	119	−0.142
Methanol (mg/L)	9.06	0.92	0.181
BOD_in_ (mg/L)	146	13	0.320 *
COD_in_ (mg/L)	296	24	0.522 **
Ammonium_in_ (mg-N/L)	28.17	2.08	0.700 **
TN_in_ (mg-N/L)	29.36	1.68	0.635 **
BOD_eff_ (mg/L)	7.66	2.22	0.660 **
COD_eff_ (mg/L)	27.3	5.66	0.656 **
Ammonium_eff_ (mg-N/L)	0.18	0.08	0.355 *
TN_eff_ (mg-N/L)	9.12	0.81	−

in, influent; eff, effluent; TN, total nitrogen; * indicates *p* < 0.05, ** indicates *p* < 0.01.

**Table 4:** Average denitrifier population and Pearson’s correlation coefficient with total nitrogen concentration in secondary effluent.

Denitrifier	Average (cells/L)	Standard Deviation (cells/L)	Pearson’s Correlation Coefficients with TN_eff_
*Thauera*	2.86 × 10^12^	2.24 × 10^12^	−0.793 **
*Paracoccus*	1.65 × 10^10^	7.92 × 10^9^	−0.543 **
*Pseudomonas*-like	3.61 × 10^9^	2.65 × 10^9^	−0.404 **
Total Bacteria	1.80 × 10^13^	3.90 × 10^12^	−0.352 *
*nar*G-like	2.10 × 10^11^	8.00 × 10^10^	−0.663 **
*nap*A	5.14 × 10^9^	2.72 × 10^9^	−0.643 **

TN_eff_, total nitrogen in secondary effluent; * indicates *p* < 0.05, ** indicates *p* < 0.01.

**Table 5:** Pearson’s correlation coefficients between denitrifying bacteria and functional genes.

Denitrifier	*Thauera* spp.	*Paracoccus* spp.	*Pseudomonas* spp.-like	Total Bacteria	*nar*G-like	*nap*A
*Thauera* spp.		0.823 **	0.842 **	0.372 *	0.891 **	0.890 **
*Paracoccus* spp.			0.838 **	0.632 **	0.868 **	0.860 **
*Pseudomonas* spp.-like				0.426 **	0.815 **	0.845 **
Total Bacteria					0.426 **	0.424 **
*nar*G-like						0.852 **

* indicates *p* < 0.05, ** indicates *p* < 0.01.

**Table 6:** Pearson’s correlation coefficients between denitrifier abundance with operational conditions and rank of operational conditions in importance as determined by the redundancy analysis (RDA) forward selection method.

Pearson’s Correlation Coefficients							RDA Forward Selection		
Variable	*Thauera* spp.	*Paracoccus* spp.	*Pseudomonas* spp.-like	Total bacteria	*nar*G-like	*nap*A	Ranking	*p*	*F*
Temperature	0.739 **	0.705 **	0.462 **	0.201	0.669 **	0.796 **	**1**	**0.002**	**34.25**
pH	−0.507 **	−0.410 **	−0.479 **	−0.203	−0.486 **	−0.421 **	16	0.866	0.29
DO_anoxic_	−0.600 **	−0.674 **	−0.700 **	−0.190	−0.675 **	−0.636 **	**3**	**0.002**	**10.77**
DO_aerobic_	0.228	0.003	0.158	−0.167	0.164	0.163	**9**	**0.036**	**3.56**
SRT	0.532 **	0.619 **	0.462 **	0.306	0.652 **	0.586 **	11	0.14	1.68
HRT	0.393 *	0.190	0.551 **	−0.207	0.270	0.413**	10	0.056	3.24
MLSS	0.039	0.171	−0.038	0.310	0.383 *	−0.009	**7**	**0.008**	**5.35**
Methanol	0.183	0.149	0.499 **	−0.110	0.108	0.181	15	0.734	0.41
BOD_in_	0.000	0.191	0.274	0.073	0.125	−0.013	**5**	**0.002**	**8.66**
COD_in_	−0.327 *	−0.035	−0.009	0.066	−0.117	−0.359 *	**8**	**0.018**	**4.74**
Ammonium_in_	−0.702 **	−0.276	−0.390 *	−0.014	−0.439 **	−0.646 **	**2**	**0.004**	**13.48**
TN_in_	−0.609 **	−0.160	−0.259	0.075	−0.331 *	−0.563 **	**4**	**0.002**	**10.6**
F/M	−0.583 **	−0.258	−0.323 *	−0.014	−0.509 **	−0.596 **	12	0.232	1.38
COD/TN	0.205	0.155	0.324 *	0.030	0.226	0.092	13	0.338	1
BOD/COD	0.442 **	0.328 *	0.423 **	−0.004	0.336 *	0.473 **	14	0.526	0.69
Rs /X-N	−0.582 **	−0.285	−0.207	−0.182	−0.596 **	−0.508 **	**6**	**0.002**	**7**

* indicates *p* < 0.05, ** indicates *p* < 0.01. Via RDA forward selection, statistically significant (*p* < 0.05). environmental variables involved in the final RDA were indicated in bold.
